# Advancing intraoperative cerebral blood flow monitoring: integrating imaging photoplethysmography and laser speckle contrast imaging in neurosurgery

**DOI:** 10.1007/s12200-025-00163-5

**Published:** 2025-09-26

**Authors:** Alexei A. Kamshilin, Anton N. Konovalov, Fyodor V. Grebenev, Igor O. Kozlov, Dmitry D. Stavtsev, Gennadii A. Piavchenko, Ervin Nippolainen, Valeriy V. Zaytsev, Alexey Y. Sokolov, Dmitry V. Telyshev, Sergei L. Kuznetsov, Roman V. Romashko, Igor V. Meglinski

**Affiliations:** 1 Institute of Automation and Control Processes, Far East Branch of the Russian Academy of Sciences, Vladivostok, 690041 Russian Federation; 2 N. N. Burdenko National Medical Research Center of Neurosurgery, Moscow, 125047 Russian Federation; 3 I. M. Sechenov First Moscow State Medical University, Moscow, 119991 Russian Federation; 4 National Research University of Electronic Technology - MIET, Zelenograd, Moscow, 124498 Russian Federation; 5 I. P. Pavlov Institute of Physiology, Russian Academy of Sciences, St. Petersburg, 199034 Russian Federation; 6 I. P. Pavlov First St. Petersburg State Medical University, St. Petersburg, 197022 Russian Federation; 7 College of Engineering and Physical Sciences, Aston University, Birmingham, B47ET UK

**Keywords:** Neurosurgery, Cerebral perfusion monitoring, Blood flow visualization, Imaging photoplethysmography, Laser speckle contrast imaging

## Abstract

Intraoperative assessment of cerebral hemodynamics is crucial for the success of neurosurgical interventions. This study evaluates the potential of laser speckle contrast imaging (LSCI) and imaging photoplethysmography (IPPG) for contactless perfusion monitoring during neurosurgery. Despite similarities in their hardware requirements, these techniques rely on fundamentally different principles: light scattering for LSCI and light absorption for IPPG. Comparative experiments were conducted using animals (rats) when assessing the reaction of cerebral hemodynamics to adenosine triphosphate infusion. The results show different spatial and temporal characteristics of the techniques: LSCI predominantly visualizes blood flow in large venous vessels, especially in the sagittal and transverse sinuses, showing a pronounced modulation associated with the heart that cannot be explained by venous blood flow alone. In contrast, IPPG quantifies the dynamics of perfusion changes in the parenchyma, showing minimal signal in large venous vessels. We propose that LSCI signal modulation is significantly influenced by the movement of vessel walls in response to mechanical pressure waves propagating through the parenchyma from nearby arteries. A novel algorithm for LSCI data processing was developed based on this interpretation, producing perfusion indices that align well with IPPG measurements. This study demonstrates that the complementary nature of these techniques (LSCI is sensitive to blood cells displacements, while IPPG detects a change in their density) makes their combined application particularly valuable for comprehensive assessment of cerebral hemodynamics during neurosurgery.

**Graphical Abstract:**

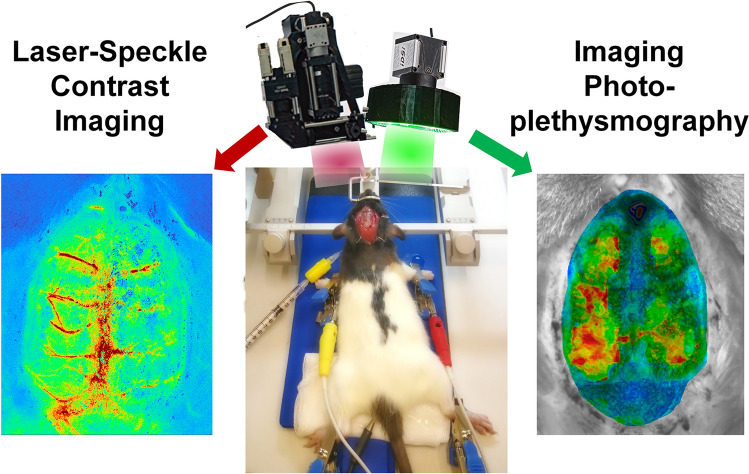

## Introduction

Modern neurosurgery has a huge technical potential that allows performing operations of almost any degree of complexity. Obviously, the success of surgical treatment strongly depends on how well blood flow and patency of the cerebral vessels are evaluated during the surgery. However, an objective intraoperative assessment of changes in regional blood supply to the brain remains an unsolved task. This forces us to continue searching for new methods that can improve the safety of neurosurgical operations and identify possible adverse blood supply rearrangements as early as possible (during surgery) to eliminate them. The requirements for the measurement/visualization system are very tough: it should be easy to handle, contactless, and provide quantitative visualization of blood flow in a wide field of view in real time with high spatial and temporal resolution. It is also important to evaluate changes in cortical blood flow, refraining from administering any fluorescent agent throughout the intervention.

Optical methods are considered as very promising for non-contact measurements of blood flow parameters. Among the wide variety of these methods, laser speckle contrast imaging (LSCI) and imaging photoplethysmography (IPPG) stand out due to the extreme simplicity of their technical implementation and the ability to visualize blood flow in full field of view. Recently, a number of pilot studies were reported demonstrating the prospects of both LSCI [1–6] and IPPG [7–9] for intraoperative assessment of cortical blood flow during neurosurgery. To assess changes in blood flow parameters using either of these techniques, only a standard digital camera to video properly illuminated tissue under study and processing software are required. In spite of similarities in the hardware implementation of the techniques, they differ significantly in the essence of the blood flow parameters they reveal. While it is necessary to use a coherent laser beam in the LSCI method, the IPPG operates by illuminating the tissue with incoherent light. The basic principle behind the both techniques is light intensity modulation caused by vascular changes. The fundamental difference between these methods is that the main cause of light modulation in LSCI is light scattering [10, 11], whereas in IPPG it is light absorption [12, 13]. Despite long-term and extensive research of both techniques, the details of the mechanisms responsible for formation both LSCI and IPPG waveforms remain fraught with uncertainty.

## Methods

### Animals

All experiments were carried out according to the ethical guidelines of the International Association for the Study of Pain, the Directive 2010/63/EU of the European Parliament and the Council on the protection of animals used for scientific purposes, and reported in compliance with the ARRIVE guidelines 2.0. Three one-month old male Long Evans rats (bodyweight 130–170 g) obtained from Institute of Bioorganic Chemistry Vivarium (Moscow, Russia) were used for the experiments. Animals were kept in the vivarium of the Sechenov University for two weeks quarantine period under the supervision of a veterinarian. Animals received water and feeding ad libitum. Storage of the animals was carried out with temperature and humidity control with 12 h day-night light cycle. The study was approved by the Sechenov University local ethics committee, protocol Nº 23–22, November 17, 2022.

### Anesthesia and surgical preparation

**Fig. 1:**
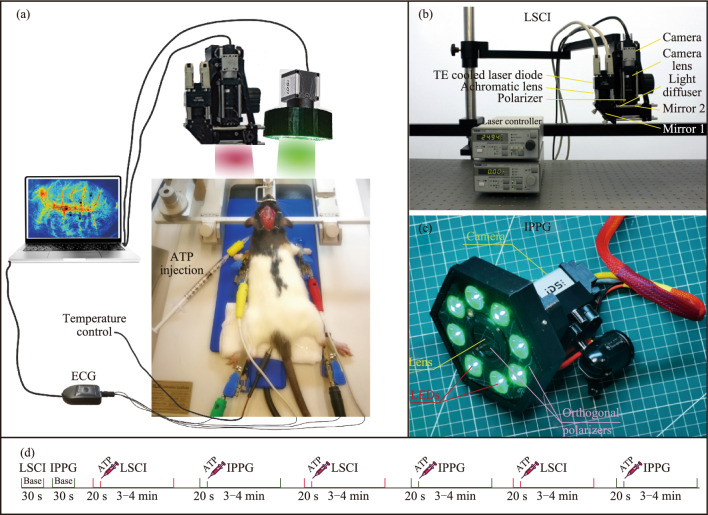
Schematics of the setup for assessing cerebral blood flow by laser speckle contrast imaging (LSCI) and imaging photoplethysmography (IPPG). **a** LSCI and IPPG systems are used sequentially to record brain images through a closed cranial window, the video sequences of which are transmitted to a personal computer. An electrocardiogram (ECG) is simultaneously recorded to the computer. **b** In-house made LSCI system. **c** In-house made IPPG system. **d** An example of the flow diagram of the experimental protocol

At the beginning of the experiment the animals were anesthetized by intramuscular injection of a combined tiletamine/zolazepam solution (“Zoletil”, Vibrac, France) in a dose of 20 mg/kg and xylazine (“Xyla”, Interchemie, the Netherlands) in a dose of 5 mg/kg. After shaving the surgical field, the animals underwent catheterization into the right external jugular vein [14] for further administration of ATP (“Sodium Adenosine Triphosphate”, Ellara, Russia) at a dose of 1 mg/kg. After detachment soft tissues of the skull and subsequent thinning the parietal bones with a micro-drill to the state of a thin membrane, the animals were placed in a stereotaxic apparatus (RWD, China), as shown in Fig. 1a. Throughout the experiment, the rat was placed on a temperature-controlled heating pad, ensuring a constant body temperature of 37°C.

### Experimental protocol

Figure 1 shows the layout of an experimental setup to measure the response of cerebral hemodynamics in rats to administration of ATP. Since LSCI requires brain illumination with coherent light at wavelength of 785 nm, but IPPG with incoherent light at 525 nm, response measurements were taken sequentially to eliminate interference from one system to another. A typical timeline of the experimental protocol, which began with 30-s baseline recordings by one system and the other, is shown in Fig. 1d. Then, in each session, video recording of the rat's brain by means of one of the systems began approximately 15 s before ATP administration and lasted continuously for 3 − 4 min. Thereafter, we replaced the measurement system (which took 1–2 min) and started a new session with the next injection of ATP. If for one rat the first session was recorded using LSCI, then for the other rat it was recorded using IPPG. All video recordings were performed simultaneously with ECG recordings. The protocol was applied to three rats, for a total of 9 sessions of ATP administration recorded using LSCI and 9 using IPPG.

### Measuring systems

#### Hardware description of LSCI

The LSCI system included digital camera acA2040-90umNIR (Basler AG, Germany) with sensitivity increased in the near infrared region and equipped by the camera lens MVL50M23 (Thorlabs Inc., USA). Illumination of the closed cranial windows (CCW) was provided by the laser light source LD785-SH300 with 300-mW maximal output power emitting at the wavelength of 785 nm. The laser was mounted into a temperature-controlled laser diode holder. Control of the current and temperature was performed by LDC200CV and TED200C devices (Thorlabs Inc., USA), respectively. The radiation from the laser was focused using an aspherical lens (C060TMD-B, Thorlabs Inc., USA) and directed to the object through an ED1-C20 light scatterer (Thorlabs Inc., USA) using two movable dielectric mirrors as shown in Fig. 1b. The CCW images with the resolution 2048 × 2048 pixels were recorded at the rate of 33.3 frames per second (fps) with 1000 μs exposure time.

#### Processing of LSCI data

According to the widely accepted theory behind LSCI [15, 16], a contrast value of speckles ${K}^{2}$, defined as the square of ratio of standard deviation σ to mean intensity $\langle I\rangle$, is inversely proportional to velocity of particles scattering the coherent light:

1 $${K}^{2}={\left({\sigma }_{N\times N}/{\langle I\rangle }_{N\times N}\right)}^{2}.$$

Here, *N* × *N* sliding window was used to calculate the speckle contrast ${K}^{2}$. To visualize spatial distribution of the blood flow in the cortex, raw speckle images were convolved with the sliding window of *N* = 5. For assessing the dynamics of cerebral blood flow, two regions of interest (ROI) were selected, one in the right, and another in the left hemisphere sizing 40 × 40 pixels or 0.36 × 0.36 mm^2^. The value of the spatial speckle contrast *K* was calculated using Eq. (1) and spatial averaging.

#### Hardware description of IPPG

Design of an IPPG system was similar to that described in our previous paper [17]. We used a custom-made IPPG system in which recording of the images was synchronized with a digital electrocardiograph (model KAP-01-“Kardiotekhnika-EKG”, Incart Ltd., St. Petersburg, Russia). The electrocardiogram (ECG) was recorded at a sampling rate of 1 kHz. An IPPG module for recording of rat’s brain images consists of a digital monochrome CMOS camera (8-bit model GigE uEye UI-5220SE-M-GL of the Imaging Development Systems GmbH) and an illuminator with 8 light-emitting diodes (LEDs, model TDS-P003L4F07, TDS Lighting Co., China), as shown in Fig. 1c. To provide uniform illumination of the CCW, the LEDs emitting at the wavelength of 525 ± 25 nm, flux 100 lm and power 3 W per diode were assembled around the camera lens (mode M1214-MP2, Computar, Japan) with a focal length of 25 mm. The camera lens and the LEDs were covered with polarizing films with mutually orthogonal orientation, thus reducing the influence of specular reflections and motion artifacts [18]. The IPPG module has been aligned so that the normal to the CCW surface is as close to the optical axis as possible of the camera lens. This alignment allowed us to minimize the artifacts caused by the ballistocardiographic effect [19]. The video frames were recorded at 150 fps with a resolution of 752 × 480 pixels and transmitted to a personal computer via Ethernet interface in PNG format. The ECG data in three leads was also saved in the computer together with the camera synchro pulses, thus providing frame and ECG synchronization with an accuracy of 1 ms. The distance between the camera lens and the CCW was about 10 cm.

#### Processing of IPPG data

The recorded video frames of tissues were processed together with the ECG using custom software implemented on the MATLAB^®^ platform. At the first step, the digital stabilization of the tissue images was applied using an optical flow algorithm with floating time window to minimize the influence of motion artifacts [20]. Taking into account that different parts of the tissue image change stochastically and heterogeneously, each image was divided into 16 × 16 pixels segments with estimation and subsequent compensation of every segment motion independently. The duration of the floating time window for image stabilization was chosen to be equal to the cardiac cycle, determined by the position of the R-peaks of the synchronously recorded ECG. All subsequent calculations were performed after subtracting motion-related components from the image averaged within the floating time window. At the second step, a PPG waveform was calculated as a frame-by-frame evolution of every pixel value. Considering that the PPG waveform consists of an alternating component (*AC*), fast-varying at the heartbeat frequency, and a slowly varying component (*DC*), which both are directly proportional to the intensity of the incident light [21], we calculated *AC*/*DC* ratio to compensate unevenness of illumination. At the third stage, the normalized amplitude of the pulsatile component (*APC*) was mapped as the amplitude of the *AC/DC* oscillations during each cardiac cycle, the beginning and end of which were determined by the ECG. At the last step, we selected two ROIs in the right and left hemispheres of the rat cerebral cortex, the position and size of which were the same as in the LSCI maps. In each of these ROIs, a perfusion index ${Perf}_{IPPG}$ was calculated as following:

2 $${Perf}_{IPPG}=\overline{APC }/\Delta CC,$$

where $\overline{APC }$ is the pulsation amplitude averaged over all pixels within each ROI, and $\Delta CC$ is the duration of the cardiac cycle over which *APC* is calculated. In every cardiac cycle, the main force driving red blood cells (RBC) is the difference between systolic and diastolic pressure. The greater this difference, the more RBC will move during the cardiac cycle. According to the recently proposed model of the IPPG signal formation [22]. *APC* is proportional to the systolic-diastolic pressure difference. Consequently, the ${Perf}_{IPPG}$ parameter defined in Eq. (2) allows tissue perfusion to be assessed in relative units proportional to blood volume per second. Calculating the index ${Perf}_{IPPG}$ in each cardiac cycle allows us to evaluate the dynamics of cerebral perfusion in response to ATP infusion in any selected area of the cortex.

## Results

**Fig. 2:**
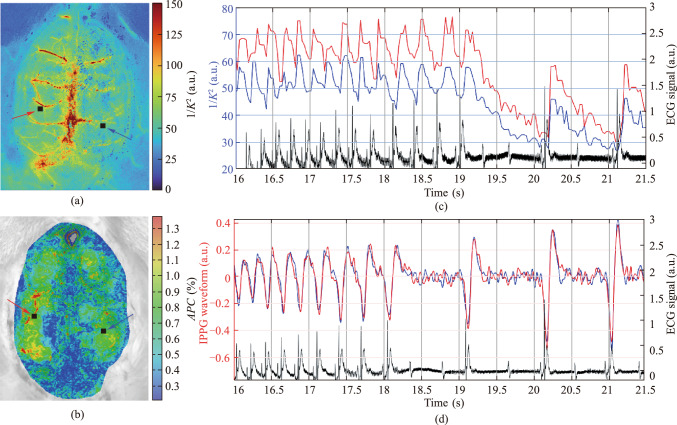
**a** Spatial distribution of the inversed speckle contrast ($1/{K}^{2}$) obtained by LSCI. **b** Spatial distribution of the amplitude of pulsatile component (*APC*) measured by IPPG. The distributions were assessed in consecutive sessions of ATP infusion for the same animal. **c** Time course of speckle contrast changes in two small regions of interest (ROIs) selected near veins in the right (red trace) and left (blue trace) hemispheres, measured with LSCI during the first 5 s after ATP administration. **d** The PPG waveforms, estimated in the same ROIs using the IPPG system. The locations of the selected ROIs are shown in **a** and **b** with black squares and highlighted with red and blue arrows. Black curves in **c** and **d** show simultaneously recorded electrocardiograms

Typical examples of the spatial distribution of blood flow parameters over cerebral cortex computed using LSCI and IPPG systems are shown in Fig. 2a and b, respectively. These distributions were obtained for the same rat in two consecutive sessions of ATP administration: the session recorded by the LSCI system was the 3d ATP infusion for this rat, while the 4th infusion was recorded by the IPPG system. Both blood-flow parameters mappings were assessed at 10th seconds after the start of each session. It is clearly seen that the distributions are radically different from each other. LSCI visualizes blood flow in larger vessels, predominantly veins, with the most intense flow observed in the sagittal and transverse sinuses, which is consistent with the observations of other groups [14, 23–26]. On the contrary, blood pulsations detected in large venous vessels using IPPG system are significantly smaller than in other cortex areas.

Dynamics of changes in speckle contrast assessed using LSCI in two small ROIs during the first 5 s after ATP infusion are shown in Fig. 2c, and IPPG waveforms measured in ROIs of the same size and location over a similar time interval are shown in Fig. 2d. The black curves in Fig. 2c and 2d show the ECG time-traces recorded synchronously with the optical data in the respective sessions of the ATP infusion. In both cases, according to ECG data, the administration of ATP initially led to a gradual decrease in heartrate observed from 17 to 19 s, and then severe bradycardia began. It is worth noting that both LSCI and IPPG waveforms have a pronounced modulation over time, strongly correlating with heartrate. As can be seen in Fig. 2, every local minimum in both waveforms occurs with a certain (almost constant) delay after each ECG R-peak, despite the significant variance in the duration of heartbeats.

**Fig. 3:**
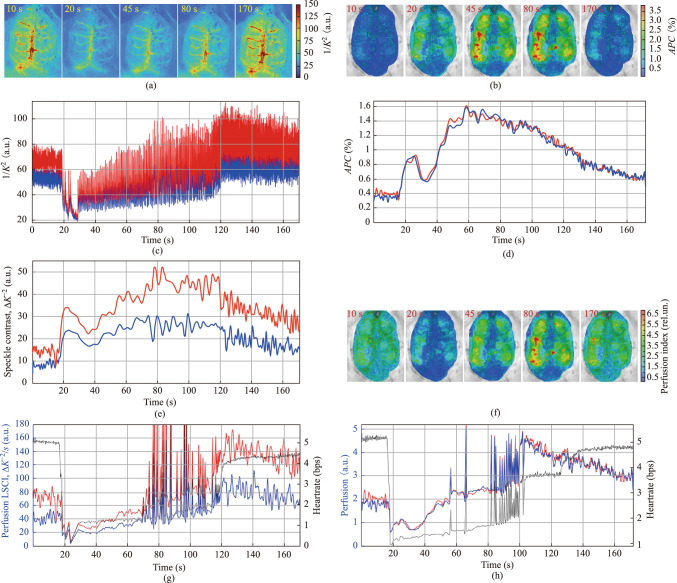
Comparison of hemodynamic changes in response to ATP infusion, assessed using LSCI and IPPG. **a** Sequence of images with spatial distribution of inversed speckle contrast computed during ATP infusion at different time points using LSCI. The color scale of the contrast (shown to the right of the panel) is the same for all five mappings in the row. **b** Sequence of images with spatial distribution of the amplitude of the pulsatile component (*APC*) acquired using IPPG at similar time points for the same animal in response to the next ATP infusion (following the one shown in **a**). **c** Speckle contrast dynamics assessed using LSCI throughout the entire ATP infusion session in two small ROIs, the position of which is shown in Fig. 2a. **d** *APC* dynamics, assessed using IPPG in the subsequent ATP infusion session to the same animal in the ROIs of the same size and location as in the previous session. **e** Dynamics of the amplitude of the pulsatile component of the speckle contrast, calculated using Eq. (3) for the same ATP infusion session as in **a** and **c**. **f** Sequence of images with spatial distribution of the cerebral perfusion index calculated using Eq. (2) at the same time points as in **b**. **g** Dynamics of the cortical perfusion assessed using LSCI and calculated with Eq. (4). **h** Dynamics of the cortical perfusion assessed using IPPG and calculated with Eq. (2). Black curves in **g** and **h** show the response of heartrate to ATP infusion in the consecutive sessions

Evolution of the spatial distribution of the inversed speckle contrast in response to ATP administration, acquired using LSCI at five characteristic time points, is shown in Fig. 3a. Comparing the LSCI maps acquired at 10-th and 20-th seconds in Fig. 3a, one can see that the inversed speckle contrast drops down immediately after ATP administration, which is usually interpreted as a sharp decrease in the RBC speed over the whole field of observation. Such dynamics of blood flow parameters is radically different from what was assessed in the subsequent session using the IPPG system, where a significant increase in *APC* mapping was observed (see Fig. 3b).

The difference between the blood flow parameters assessed using LSCI and IPPG becomes even clearer if we compare the dynamics obtained during two consecutive ATP infusion sessions (compare Fig. 3c and Fig. 3d). The dynamics of the inverted speckle-contrast assessed in two small ROIs marked with black squares in Fig. 2a, is presented in Fig. 3c. *APC* time-traces estimated throughout the subsequent session using IPPG in the ROIs of the same size and location as in the previous session (see Fig. 2b) are shown in Fig. 3d. As clearly seen in Fig. 3c, the amplitude of the LSCI signal modulation at heartrate is changing over time during the session.

To elucidate the character of these changes, we calculated the modulation amplitude $\Delta K$ as the difference between the maximal and minimal value of the inversed speckle contrast:

3 $$\Delta K={\left(1/{K}^{2}\right)}_{\text{max}}-{\left(1/{K}^{2}\right)}_{\text{min}}.$$

Here, ${K}^{2}$ is the speckle contrast defined in Eq. (1), while the maximum and minimum of the LSCI waveform are estimated within every cardiac cycle, the beginning and end of which are determined by the R-peaks of the simultaneously recorded ECG. The time-traces of $\Delta K$ calculated for the LSCI waveforms shown in Fig. 3c are presented in Fig. 3e. Evidently, the pattern of changes in the parameter ∆*K* due to ATP infusion (Fig. 3e), now looks very similar to the pattern of changes in *APC* caused by subsequent ATP infusion (Fig. 3d).

It should be noted that in all 18 sessions of ATP infusion, approximately 4 s after drug administration, a multiple (6 − 11 times) decrease in the heartrate was observed in ECG, which can be characterized as severe bradycardia. This effect is typical for ATP infusion [27]. An example of such a decrease can be seen in Fig. 3g, where the response of the heartrate to the ATP infusion is presented by the black curve. According to both IPPG and LSCI data, a sharp drop in the heartrate is accompanied by a simultaneous significant increase in the pulsation amplitude of both waveforms (see Fig. 3d and Fig. 3e). Since the heart-related pulsations amplitude in cerebral tissues, quantified by *APC*, has been shown to be inversely proportional to the vascular tone of the arteries supplying the cortex [17, 28, 29], the observed increase in *APC* may be associated with an attempt by the cerebrovascular autoregulation system to compensate for delays in brain perfusion.

As we pointed out in Sect. 2.4.4, the pulsation amplitude itself can be used as a perfusion index only if the duration of the cardiac cycle remains unchanged. Since this condition is not met when ATP is administered, the perfusion index in IPPG measurements was calculated using Eq. (2), which normalizes *APC* for the cardiac cycle duration. Perfusion index maps overlaid with raw images of rat cortex, and assessed by IPPG at the same representative time points as in Fig. 3b are shown in Fig. 3f. As one can see, spatial distributions of the perfusion index shown in Fig. 3f vary over time in a different way than the spatial distributions of *APC* shown in Fig. 3b. The dynamics of the cortical perfusion index, estimated using IPPG in the same ROIs as the time dependences of blood flow parameters in Fig. 3c, d, and e, and calculated according to Eq. (2), is shown in Fig. 3h. It can be seen that at the beginning of severe bradycardia, perfusion drops sharply, but then gradually recovers. It took about 30 s to restore perfusion, followed by hyperperfusion, which reached its maximum 90 s after the start of the ATP infusion.

For calculation of the perfusion index in the case of the LSCI measurement, we used a similar approach, normalizing $\Delta K$ to the duration of the cardiac cycle:

4 $${Perf}_{LSCI}=\Delta K/\Delta CC,$$

where $\Delta K$ is the pulsatile amplitude assessed by LSCI in every heartbeat, and $\Delta CC$ is the duration of the respective cardiac cycle. Changes in ${Perf}_{LSCI}$ in response to ATP infusion estimated using LSCI in the same ROIs is shown in Fig. 3g. As seen, the pattern of cortical perfusion changes is similar to that observed by using IPPG (Fig. 3h). However, the perfusion is recovered after 50 s, and the maximum of reperfusion as reached at 105 s after the start of the ATP infusion.

Each of the three rats received six intravenous injections of ATP at intervals from 5 to 7 min. Cerebral hemodynamics was measured by different imaging techniques alternately: in one rat, first by the IPPG method, then by the LSCI method, and so on, in another rat, measurements were carried out in reverse order. In all experimental sessions, both systems revealed a similar pattern of changes in cerebral perfusion in response to ATP administration only if the perfusion index was assessed as the amplitude of pulsations of optical signals normalized to the duration of the cardiac cycle in accordance with Eq. (2) or Eq. (4). Approximately five seconds after the start of ATP administration, a sharp decrease in heartrate was observed, accompanied by a drop in the perfusion index. This was followed by a gradual recovery of cortical perfusion, which developed into hyperperfusion, exceeding the baseline values by 2.70 ± 0.83 times. On average for experimental sessions, perfusion index reached its maximum value at 108 ± 33 s after the start of the ATP infusion.

A common feature of all observations of perfusion dynamics, assessed using both IPPG and LSCI, is a multiple increase in the amplitude of optical signal pulsations caused by ATP administration, which significantly exceeds the corresponding increase in the perfusion index: 3.87 ± 1.26 versus 2.70 ± 0.83 times (*P* = 0.01). Moreover, the maximum value of the perfusion index is achieved 26 ± 12 s later than the maximum value of the pulsation amplitude (cf. Fig 3e with Fig. 3g or Fig. 3f with Fig. 3h). The duration of both severe bradycardia and the recovery period varies between each ATP injection and from one animal to another.

It is worth noting that thinning the cranial bone of one of the animals using an automated polishing system led to an insufficiently transparent cranial window, which did not allow us to visualize the cerebral blood flow in this rat using LSCI. Nevertheless, hemodynamics parameters were successfully assessed in this rat using the IPPG system. The preparation of the closed cranial window in two other animals was done manually of a higher quality, which allowed us to visualize and assess the parameters of cerebral blood flow using both IPPG and LSCI.

## Discussion

Our experimental study has shown that both optical systems do provide visualization of cerebral blood-flow parameters in full field of view and reveal change in hemodynamics caused by ATP administration. In all studies using the LSCI method, the reverse speckle contrast is considered the main quantitative index for assessing changes in cerebral blood flow [1, 30–32]. This approach is based on the hypothesis that light scattering from RBCs moving through vessels leads to a significant decrease in speckle contrast (a greater speckle decorrelation) than occurs with scattering on slower RBCs. Despite a number of reports that venous vessels exhibit both significant decorrelation of speckle contrast [14, 23–26], and strong modulation of the LSCI waveform at heartrate [23, 25, 33], not much attention has been paid to the explanation and interpretation of these observations.

In fact, the observed correlation between speckle contrast modulation and cardiac cycles in venous measurements (such as those shown in Fig. 2c) are attributed to the propagation of pressure waves through the vascular system. Although venous flow is generally considered steady, the cardiac-induced pressure waves create significant variations in blood cell velocity. These dynamic changes manifest in the laser speckle contrast signal due to temporal decorrelation of speckle patterns and alterations in scattering properties.

It should be emphasized that not every RBC displacement can be considered informative in terms of quantifying brain perfusion. The task of perfusion is to ensure the supply of blood cells to the capillary network for subsequent efficient metabolism. In this regard, only those particles that move inside and along arterial vessels should be taken into account when assessing perfusion. Since blood enters the veins after passing through a capillary network with maximum hydrodynamic resistance, which should significantly suppress the pulse wave, it is unlikely that cardiac-induced pressure wave directly results in significant motion of scattering particles, manifested as speckle decorrelation (see Fig. 2a and Fig. 3a) and observed in the region of sinuses. We believe that blood cells in the sinuses predominantly move along the vessel, but venous flow dynamics differ from arterial pulsatility. The observed speckle contrast fluctuations in LSCI suggest that venous blood flow has pulsations, probably due to mechanical interaction with surrounding structures, rather than direct propagation of cardiac pressure pulse. In the arteries, blood cells move predominantly along the vessel due to the pulse pressure wave, and the speckle decorrelation caused by this movement refers to perfusion. In a parenchyma containing smaller vessels, the change in speckle contrast is influenced by both light scattering from RBCs moving along the vessels and scattering on the oscillating walls. Therefore, the relationship between speckle decorrelation and perfusion is very complicated and depends on both the type of blood vessels and their relative location.

**Fig. 4:**
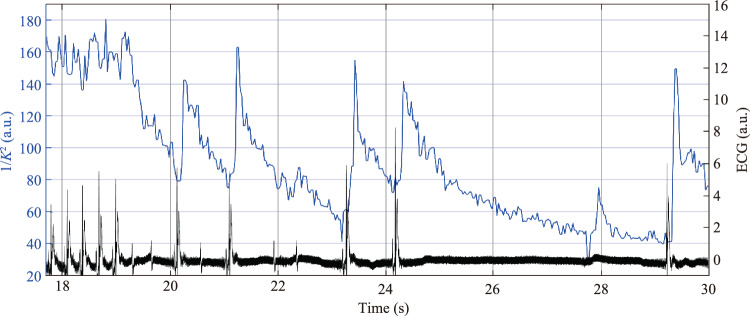
An example of the dynamics of the inversed speckle contrast assessed by the LSCI in a small ROI located in the middle of sagittal sinus

The effect of vascular wall fluctuations excited by transmural pressure waves may also explain the observed correlation between heartbeat and speckle-contrast modulation in a small ROI located in the middle of the sagittal sinus (Fig. 4). It can be seen that the inverse speckle contrast reaches a minimum with a slight delay after the ECG R-peak. This means that the velocities of the scattering patterns are minimal at this time point. Then there is a sharp increase in velocity, which raises the question: what is the reason for the appearance of a pulse wave in the sinus, the amplitude of which even increases with the expected perfusion drop caused by the bradycardia? We suggest that this velocity jump is unlikely to be associated with perfusion, but occurs due to the movement of cells perpendicular to the axis of the sagittal sinus caused by oscillations of its walls.

Recently Postnov et al. discussed speckle contrast modulation in veins [25]. They suggested that “the pulse wave in the vein is formed by: (i) the forward propagating pulse pressure wave, (ii) surrounding tissue motion and (iii) a backward propagating (“pulling-back”) cardiac wave” [25]. However, reason (iii) can hardly be used to interpret the waveform in Fig. 4, since for a pulse wave propagating backward in the veins, the pressure peak should be much more delayed relative to the R peak than is observed in our experiments [34–36]. Concerning the reason (i), we notice again that the forward propagating wave in the veins is expected to be much smaller than in arteries. So, there remains the reason (ii), which, unfortunately, was not specified in sufficient depth in previous studies [25]. Based on the results of current study we suppose that of all the diverse motions of surrounding tissues, the displacement of the walls of large blood vessels has the most significant effect on the formation of dynamic speckles.

The greatest amplitude of pressure pulsations associated with heart beats is observed in the arteries. These pulsations are naturally transmitted to the parenchyma, exciting acoustic vibrations there. These vibrations cause the boundaries of the parenchyma to move, especially on the venous walls, since the blood pressure in the veins is quite low. Light scattering on blood cells excited by oscillating walls is the reason of speckle decorrelation. The observed changes in the amplitude of oscillations of the inversed speckle contrast in response to ATP infusion and the similarity of these changes to the dynamics of IPPG (see Fig. 3d and Fig. 3e) suggest that the modulation of the contrast with heartbeats is mainly caused by the pressure pulsations in arteries, while the influence of scattering by moving RBCs along the sinuses cannot be excluded. Therefore, although changes in speckle contrast are most pronounced in the area of large sinuses (Fig. 2a and Fig. 3a), it is pulsatile arterial pressure that is the reason of speckle decorrelation in these regions.

The proposed interpretation of speckle decorrelation by pulsatile blood flow in biological tissue in vivo is an extension of the alternative model of the IPPG signal formation [22]. According to this model, it is pulse oscillation of the artery walls that transmurally compresses and releases the capillary network in the parenchyma. These acoustic waves reversibly change the distance between the capillaries, thereby modulating the density of RBCs that actively absorb green light, which ultimately modulates the intensity of the returned light. By this way, the capillary network serves as a naturally distributed transducer of the pulse waves of arterial blood pressure into changes in light intensity [22, 37]. The closer the pulsating artery is located to the cerebral cortex, the greater the amplitude of the modulation of the reflected light intensity will be observed. However, vibrations of the vascular walls do not modulate the absorption of reflected light in large vessels, since the density of RBC remains unchanged due to the incompressibility of the fluid. Since the sagittal and transverse sinuses are located on the surface of the cerebral cortex, where there is no transducer in the form of a capillary network, IPPG does not visualize pulsations in them (see Fig. 2b and 3b).

As our experimental study has shown, the waveform of both IPPG and LSCI is primarily determined by pressure pulsations in the arterial vessels. It means that the amplitude of the light-intensity modulation at heartrate (referred to as *APC* in IPPG, and $\Delta K$ in LSCI) is proportional to the difference between systolic and diastolic blood pressure in arteries supplying a local area of the cerebral cortex visible by the camera sensor. As it was shown, the *APC* parameter in the IPPG system is a measure of cerebral vascular tone [17, 28]. Based on the similar dynamics of changes in $\Delta K$ and *APC* (Fig. 3d and e) in response to ATP administration, we hypothesize that the $\Delta K$ parameter (as opposed to speckle contrast) is also a marker of vascular tone, which determines the magnitude of the difference between systolic and diastolic blood pressure. Additional experiments and/or simulations could further support our hypothesis.

Since during every cardiac cycle, the difference in systolic and diastolic pressure (which is proportional to either *APC* or $\Delta K$) is the only driving force for RBCs, in this study, the parameters *APC* and $\Delta K$ normalized to the cardiac cycle duration ($\Delta CC$, see Eq. (2) and Eq. (4)) were used as markers of cerebral perfusion. The dynamics of perfusion in response to ATP administration, estimated using IPPG and LSCI in our study, in all cases showed that the onset of severe bradycardia is accompanied by a multifold increase in both *APC* and $\Delta K$ (Fig. 3d and e) due to a decrease in arterial tone caused by vessels dilatation (which is the well-known effect of ATP) [38, 39]. This indicates the intention of the autoregulation system to compensate for the effect of increasing the duration of the cardiac cycle. However, after a few seconds, the relatively slowly increasing blood supply turns out to be insufficient to compensate for the loss of brain nutrition, which leads to an abrupt increase in the duration of the cardiac cycle (severe bradycardia) and, accordingly, to a sharp drop in the perfusion index, calculated as *APC* or $\Delta K$ normalized to $\Delta CC$ (Fig. 3g and h). Then, in all sessions, a gradual increase in the perfusion index is observed, which reaches its maximum after 108 ± 33 s. Hyperperfusion was many times greater (2.70 ± 0.83) than the perfusion index in baseline.

The history of using laser speckles to assess in vivo blood flow dates back to the 1980s [40, 41], and since then the main theoretical doctrine has been the model in which the main cause of speckle decorrelation is the motion of the RBCs. Without exception, all currently existing algorithms for processing LSCI data in order to obtain blood flow parameters are based on this theoretical model [30–32, 42–45]. The data obtained in the current study do not dispute this model, but emphasize that not every displacement of blood cell can be associated with perfusion. To relate the LSCI signal to perfusion, we proposed a new data processing algorithm based on the assumption that the cause of cell velocity modulation is pressure waves generating in nearby arteries, but reaching the venous walls after compression/decompression of the connective tissues of the parenchyma.

The cortical perfusion response to ATP infusion, estimated by applying the new algorithm to the LSCI data, is essentially different from that calculated using the generally accepted algorithm (cf. Fig 3c and Fig. 3e). It is astonishing that the pattern of the responses assessed using the new algorithm fits well with the pattern of responses obtained from IPPG data for all ATP infusion sessions in different animals. However, these methods visualize cortical blood flow in completely different ways (see Fig. 3a and Fig. 3b), which is a consequence of the fundamental difference between them: if the cause of light modulation in LSCI is its scattering, then in IPPG it is absorption. On the one hand, this difference gives some technical advantages to the IPPG system, since static scattering has less effect on signal generation than in LSCI. This statement has been proved in our study, which demonstrated that in an animal with a poorly polished skull bone, the LSCI signal is very noisy, whereas the IPPG system allowed us to measure the perfusion dynamics even in this animal. On the other hand, it is precisely due to the different physico-physiologic backgrounds of these systems that their combined use in assessing cerebral perfusion will be able to shed light on as yet unknown features of hemodynamics.

It is worth noting that several studies have been carried out to compare the performance of LSCI and IPPG systems in non-contact assessment of tissue perfusion [46–50]. However, these studies did not find significant differences in the visualization of blood flow detected by these systems, likely because they were all conducted on biological tissues in which it is difficult to identify blood vessels (forehead, forearm, wrist), and not on the brain.

The limitation of this study is the small number of animals studied, which was the consequence of the first attempt to experimentally compare two most promising techniques capable for intraoperative monitoring of cortical blood flow parameters. The observed difference in the responses of cortical blood flow parameters to the same pharmacologic impact measured by two basically different techniques turned out to be so significant and repeatable that we considered it necessary to bring these results up for discussion. Our near future plans include carrying out comparative studies of IPPG and LSCI on a much larger sample together with simultaneous monitoring of the main physiologic parameters, primarily a simultaneous assessment of the systemic blood pressure.

## Conclusion

This study presents significant findings in cerebral blood flow monitoring through optical imaging techniques. Our research demonstrates that conventional LSCI data processing algorithms, while effectively visualizing blood flow in large venous vessels, produce signal modulations that are challenging to explain physiologically. The observed strong cardiac-related modulation in venous vessels suggests that the LSCI signal formation mechanism is more complex than previously thought. Our newly proposed interpretation and processing algorithm, which considers the contribution of vessel wall movements to speckle decorrelation, provides results that align well with IPPG measurements from the same animal subjects.

Both LSCI and IPPG systems demonstrate considerable potential for intraoperative cortical blood flow monitoring, offering distinct and complementary views of cerebral hemodynamics. While LSCI predominantly visualizes large venous vessels through light scattering, IPPG reveals blood pulsations in the parenchyma through light absorption. This fundamental difference in their underlying mechanisms and anatomical targets suggests that their combined use could provide more comprehensive and reliable intraoperative hemodynamic analysis than either technique alone.

The simplicity of both systems’ implementation—requiring only standard cameras and appropriate lighting, with no need for contrast agents – makes them particularly suitable for clinical applications. Their ability to provide accurate visualization of cortical perfusion responses across multiple spatial and temporal scales further enhances their practical utility. Future research investigating the integrated application of these complementary techniques during various neurosurgical interventions could help establish optimal protocols for intraoperative cerebral blood flow monitoring, potentially leading to improved surgical outcomes through enhanced real-time assessment of cortical perfusion.

## Data Availability

The data that support the findings of this study are available from the corresponding author, upon reasonable request.
